# A Growing Triple Burden of Malnutrition in South Asia Due to the Cumulative Effect of Double Burden of Malnutrition and Parasitic Infections in South Asian Low- and Middle-Income Countries: A Scoping Review

**DOI:** 10.3390/nu17213494

**Published:** 2025-11-06

**Authors:** Rameshwor Parajuli, Wilna Oldewage-Theron

**Affiliations:** 1 Central Department of Zoology, Tribhuvan University, Kirtipur 44613, Nepal; rameshwor.parajuli1@gmail.com; 2 Innovative Agro-Processing for Climate-Smart Food Systems, University of the Free State, Bloemfontein 9300, South Africa

**Keywords:** double burden of malnutrition, infections, low- and middle-income countries, overweight and obesity, parasites, prevalence, underweight

## Abstract

**Background and Aims**: In recent decades, lifestyle patterns have undergone significant transformations, particularly in low- and middle-income countries (LMICs). These changes have contributed to a dual nutritional crisis characterized by the coexistence of undernutrition and overweight/obesity, commonly referred to as the Double Burden of Malnutrition (DBM). Compounding this issue is the persistent prevalence of parasitic infections, due to poor personal hygiene and sanitation practices which further exacerbate nutritional imbalances, creating what is now recognized as the Triple Burden of Malnutrition (TBM). This review aims to explore the evolving lifestyle factors that have contributed to the emergence of the DBM and to examine its intersection with parasitic infections. The focus is particularly on South Asian low- and middle-income countries, where these overlapping burdens present a significant public health challenge. By highlighting the interconnectedness of malnutrition, obesity, and parasitic diseases, this study seeks to provide a comprehensive understanding of the current nutritional landscape in South Asian LMICs and to inform future health interventions and policies. **Methods**: This study was conducted using published and unpublished secondary data that are available on websites and other printed materials. One of the main requirements is date, with 2013 being regarded as the initiative’s landmark. Another crucial factor is the availability of the entire article. For this study, only research publications published in English were taken into consideration. Zotero was used for compilation. The majority of the analysis was performed using percentages and ratios. A thorough evaluation of all the studies’ methodology, design, execution, and reporting was performed in order to spot any systematic flaws in this study. **Results**: Only 45 of the 105 full-text papers that were screened met the requirements for inclusion. Of these studies, 15 satisfied the inclusion and exclusion requirements. The results show that China, with a comparatively higher income level status, has more prevalence of overweight and obesity among children (11.5%) and women (34.6%) than India (2.1% of OWOB among children and 20.6% among women). Nepal stands behind China and India with 1.2% of OWOB among children and between them with 22.2% OWOB among women. Interestingly, among the three South Asian nations, India has the highest stunting, wasting, and underweight among children (38.4%, 21%, and 35.7%, respectively) followed by Nepal (35.8%, 9.7%, and 27%) and China (8.1%, 2%, and 2.5%). This study finds no significant difference in the prevalence of intestinal parasitic infections among OWOB and underweight populations. This review finds that the DBM along with parasitic infections has resulted in a Triple Burden of Malnutrition, which is currently a major public health issue in low- and middle-income countries in South Asia. **Discussion**: The various types of malnutrition were once thought of and treated as distinct public health problems, but the new understanding is that undernutrition and overnutrition are linked, and that in order for policy solutions to be successful, double-duty measures that simultaneously address multiple dimensions must be put in place. When the DBM is combined with parasite illnesses, it becomes the Triple Burden of Malnutrition, which is the primary cause of the financial burden in LMICs. China has the worst obesity problem, yet it also has more obesity-related laws and intervention programs than India and Nepal combined. All three nations, however, have failed to stop or deal with the dramatic increase in OWOB over the last 20 years. For effective implementation and results, genetic and psychological factors must also be taken into account when developing policies and programs to tackle the obesity epidemic, undernutrition, and parasite diseases. **Conclusions**: The prevalence of the DBM has been rising globally, with South Asia seeing a faster rate of increase. A growing DBM is favorably correlated with national economic development. In South Asian LMICs, the DBM combined with parasite diseases has resulted in a Triple Burden of Malnutrition, a debilitating illness.

## 1. Introduction

The Double Burden of Malnutrition (DBM) is the coexistence of undernutrition and overnutrition (including overweight, obesity, or diet-related non-communicable diseases) within individuals, households, populations, and across the life course [1]. This phenomenon occurs within a rapidly changing global nutrition landscape, shaped by economic and income growth, urbanization, demographic changes, and globalization, which have collectively driven a substantial change in diet-related epidemiology in recent decades [2]. The global magnitude of malnutrition is significant; in 2022, the World Health Organization (WHO) reported stunting (too short for age), wasting (too thin for height), and underweight in 149 million, 45 million, and 390 million children under the age of five years old (U5), respectively. Concurrently, in the same reporting year, 2.5 billion adults were classified as overweight, with 890 million classified as obese [3]. Notably, almost half of U5 deaths that year were associated with undernutrition and these occurred predominantly in low- and middle-income countries (LMICs) such as those in South Asia. Furthermore, the prevalence of childhood overweight and obesity LMICs is increasing at a rate 30% higher than in high-income countries [4].

Historically, malnutrition research focused on undernutrition, particularly stunting and wasting. However, the World Health Organization (WHO) has recently introduced a tripartite classification of malnutrition, encompassing (1) undernutrition (including stunting, underweight, and wasting); (2) micronutrient deficiencies, often exacerbated by parasitic and other infections; and (3) overnutrition (overweight and obesity). While recent studies increasingly adopt this tripartite framework, integrated discussion of all three components, leading to the concept of the Triple Burden of Malnutrition (TBM), remains limited. The TBM represents a growing public health challenge, particularly in LMICs, including the South Asian region.

Both overnutrition and undernutrition in children are associated with an elevated risk of parasitic infections. A parasite is an organism that resides in or on a host, deriving sustenance at the host’s expense. These organisms, frequently found within the human gastrointestinal tract (GIT), can disrupt digestive processes, impair nutrient absorption, and subsequently lead to nutrient deficiencies and malnutrition. Evidence suggests that parasitic infections contribute to persistent inflammation, nutritional loss, and reduced digestion and absorption, culminating in childhood malnutrition and micronutrient deficiencies [5].

Globally, parasitic infections are recognized as a significant contributor to undernutrition in preschool-aged children, with an estimated 230 million cases (43%) in resource-limited settings [6]. Current epidemiological data indicate that children with parasitic infections exhibit a higher likelihood of experiencing stunting, wasting, underweight, and general undernutrition. Commonly reported infections include giardiasis, trichuriasis, ascariasis, and hookworm infection. A recent study in Somalia found that undernourished children had a higher prevalence of intestinal parasitic infections compared to those with overnutrition [7]. This finding is consistent with a comprehensive review by Fauziah et al. (2022) that synthesized data from 17 studies across Latin America, Southeast Asia, and Africa [8]. Furthermore, a broader consensus in the literature suggests that the association between undernutrition and parasitic infections is linked to unidentified parasites, rather than specific parasites alone. This implies that polymicrobial parasitic infections may be a major cause of undernutrition [9].

**Figure 1:**
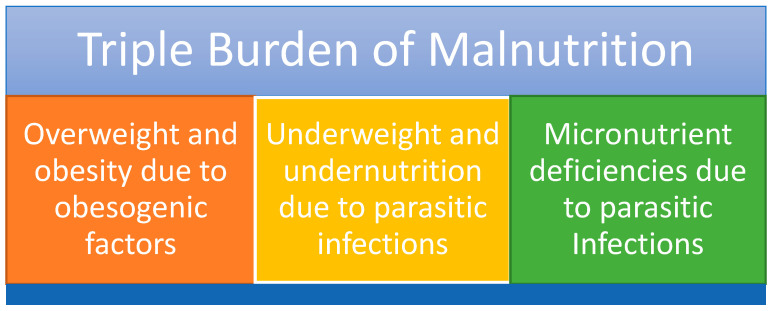
Concept of Triple Burden of Malnutrition.

Figure 1 below presents the concept of the Triple Burden of Malnutrition.

This review aims to explore the evolving lifestyle factors that have contributed to the emergence of the DBM and to examine its intersection with parasitic infections. The focus is particularly on South Asia, where these overlapping burdens present a significant public health challenge. By highlighting the interconnectedness of malnutrition, obesity, and parasitic diseases, this study seeks to comprehensively assess the current nutritional status within LMICs and to inform future health interventions and policies.

## 2. Materials and Methods

### 2.1. Study Design

A scoping review of the literature was conducted, adhering to the Preferred Reporting Items for Systematic reviews and Meta-Analyses (PRISMA). The PRISMA 2020 checklist (Table S1) was obtained from: Page MJ, McKenzie JE, Bossuyt PM, Boutron I, Hoffmann TC, Mulrow CD, et al. The PRISMA 2020 statement: an updated guideline for reporting systematic reviews. BMJ 2021;372:n71. doi: 10.1136/bmj.n71 [10]. 

**Figure 2:**
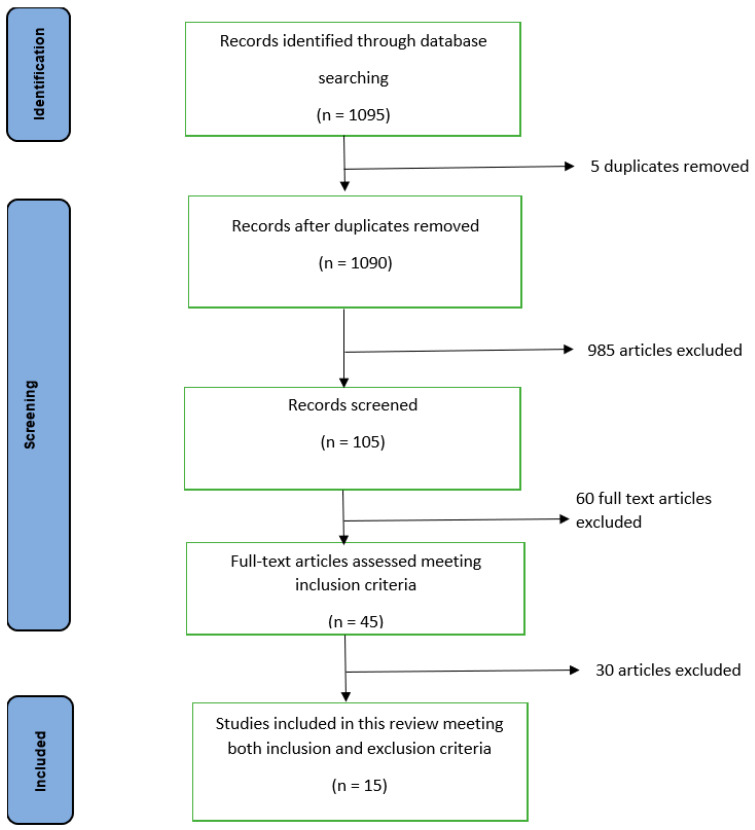
Process of identification, screening, and inclusion in this study: flow diagram.

Figure 2 below illustrates the study design with a flow diagram showing the identification, screening, and inclusion.

### 2.2. Search Strategy

We conducted a systematic literature search of the PubMed database for comprehensive and quality articles between January 2013 and June 2025 because much older articles can be irrelevant for this review. Search algorithms and keywords like “double burden of malnutrition, health burden, Gastrointestinal (GI) parasites, overweight and obesity, underweight, stunting, challenging issue, helminths infection, BMI, Intestinal Parasitic Infections (IPIs), low- and middle-income countries of South Asia, triple burden of malnutrition” were used to retrieve the relevant research. Fact sheets and survey reports from the WHO, the United Nations (UN), and the World Bank were directly extracted from their websites.

### 2.3. Exclusion and Inclusion Criteria

Date, considering 2013 as the initiative landmark, is one of the major criteria. Full article availability is another important criterion. Similarly, other important criteria considered during this review are topic matching, journal impact, and journal ranking in SJR (Scimago Journal) to obtain quality research articles. Most of the research papers in this study were from low- and middle-income countries and language barrier is one of the important criteria in this study. Only those research papers published in English were considered for this study.

### 2.4. Compilation and Synthesis

Compilation was performed on Zotero for ease of citations and references. A minimal synthesis of the results was undertaken based on ratios and percentages. Interpretation was carried out solely based on the study objectives.

### 2.5. Assessment of Risk of Bias

A thorough evaluation of all the studies’ methodology was performed to ensure the results can be trusted. In addition, the studies’ design, execution, and reporting were examined in order to spot any systematic flaws that would compromise the reliability of the results. Interpretation can be minimally wrong but there can be no interpretive bias during the interpretation.

## 3. Results

### 3.1. Search Results

The initial literature search using keywords like “double burden of malnutrition, health burden, Gastrointestinal (GI) parasites, overweight and obesity, underweight, stunting, challenging issue, helminths infection, BMI, Intestinal Parasitic Infections (IPIs), low- and middle-income countries of South Asia, triple burden of malnutrition” yielded 1095 studies, including 10 studies that were identified through reference screening.

After removing 5 duplicate articles and excluding 985 unrelated articles, as they were different from our topic and context, during title and abstract screening, 105 full-text articles were reviewed. Of these, only 45 fulfilled the inclusion criteria like full article availability and being articles of Scimago-listed journals. Out of 45 articles, only 15 ultimately met all the inclusion and exclusion criteria (publication in other language than English and date of publication; 2013 as the landmark). Figure 2 above clearly shows the identification, screening, and inclusion process of this study.

### 3.2. Results of Studies

#### 3.2.1. Global Review of IPIs of Low- and Middle-Income Countries

A Venezuelan study showed that 26.8% of households had a DBM, and these DBM-affected households were significantly associated with the presence of *Giardia lamblia* and Geohelminths [11]. This is a manifestation of the Triple Burden of Malnutrition, an important problem found in this review. A study from Peru showed that children in the home environment and in family settings thrived better than children in orphanages. The same study also showed that the prevalence of GI infections was higher in orphanages than in families [12]. A study in a rural area of Mexico found that *Entamoeba coli* infection may contribute to fat deposition [13]. The investigation also detailed the prevalence of parasitic infections: *Ascaris lumbricoides* was identified as the most prevalent Soil-Transmitted Helminth (STH) (16%) followed by hookworm. Among protozoa, *Entamoeba coli* was predominant (20%), followed by *Endolimax nana*, *Balantidium coli*, *Entamoeba histolytica/dispar*, *Iodamoeba bütschlii*, and *Giardia lamblia* [13].

A study of Ethiopian primary school students found a significant association between intestinal parasitic factors and several other factors, including children with illiterate mothers and fathers, children with fathers who could read and write, children not regularly washing their hands before meals, and a lack of awareness regarding WASH [14]. A similar study among northwest Ethiopian school children reported a high prevalence of GI parasite infections in underweight children younger than 13 years, with severe stunting also observed to be higher among girls compared to boys. Hygienic factors and literacy are associated with these parasitic infections [15]. These hygienic and literacy factors are highly prevalent in LMICs, which make them more vulnerable to undernutrition.

#### 3.2.2. Review of IPIs and DBM of China, India, and Nepal—South Asia

The prevalence of OWOB in China is notably higher than that of underweight. Large rural/urban differences are evident for both outcomes in school children and women of childbearing age [16]. Stunting is also more prevalent among rural school children compared to those in urban areas. Although gender differences for OWOB and underweight are not significant in school children [17], the prevalence of OWOB is generally higher in urban populations due to greater exposure to obesogenic factors (e.g., foods with higher energy density and a more sedentary lifestyle). An exception is observed among women of childbearing age in China, where the rural/urban OWOB is not significant. Interestingly, recent national data in China show a similar prevalence of OWOB among women of childbearing age in urban and rural areas [18]. In recent decades China’s economy has increased impressively and is now the second largest economy after the United States of America, with 10% Gross Domestic Product (GDP) growth annually [19]. Along with this economic leap, China has witnessed a considerable decline in endemic Soil-Transmitted Helminths (STHs), schistosomiasis, and malaria. However, the prevalence of some food-borne trematode infections and Cysticercosis has significantly increased in recent years [20]. *Clonorchiasis* and *Paragonomiasis* are two prominent examples of these food-borne trematode infections in China; approximately 15 million people are infected with the former nationwide [20,21]. This escalation is contributing to major health issues currently observed in China.

A recent study in India showed that being overweight or obese was linked to older age, higher education, affluence, living in an urban area, and belonging to an underperforming class [22]. A similar study in India indicated that individuals from other underperforming communities had a considerably higher likelihood of being OWOB, while a lower rate of OWOB and a higher prevalence of underweight were observed among selected tribes [23]. OWOB and underweight populations of India are at equal risk of intestinal parasitic infections in India. BMI alone is not a determinant for infection risk, but hygiene and co-morbidities are risk factors [24].

A Nepalese study, which determined the prevalence of STHs across five major communities, concluded that STH infection was associated with individual hygiene behavior (such as “not using soap for hand-washing” or “not wearing shoes outside”) [25]. However, the same study found no association between STH infection and nutritional status (being underweight or overweight) or socio-demographic characteristics (such as literacy). A similar study conducted among adolescents in Nepal further showed that individual unhygienic behaviors increase the risk of intestinal parasitic infections, and those who engage in such unsanitary behaviors are more likely to suffer from malnutrition [26]. The burden of enteric parasitic infections may be lessened by better health services and a more favorable opinion by the local population [27]. IPIs and various nutritional statuses were found to be inconsistently associated with each other in numerous previous studies. There are limited policies focusing on the prevention and control of DBM in Nepal [28] and the rapid escalation of the DBM over the past two decades remains poorly controlled. This trend is compounded by an observed lack of prevention and control efforts for key factors targeting obesogenic environments, necessitating the integration of genetic and psychological determinants into future DBM policies and programs [29].

### 3.3. Results of Syntheses

#### 3.3.1. Economic and Demographic Comparison of China, India, and Nepal

China has the highest GDP (USD 8827) among the three South Asian nations, followed by India (USD 1942.1) and Nepal (USD 849). China has the highest life expectancy (76.5 years) followed by Nepal (70.2 years) and India (68.2 years). China has the least under-5 mortalities (9.2) followed by Nepal (33.4) and India (38.7). China and India are the giant nations of South Asia, with a large population living in urban areas (58% and 33.6%, respectively).

#### 3.3.2. Prevalence of Undernutrition and OWOB Among Children in China, India, and Nepal

Stunting among children is highest in India (38.4%) followed by Nepal (35.8%) and China (8.1%). OWOB is highest in China (11.5%) followed by India (2.1%) and Nepal (1.2%). There is a huge difference in underweight children populations between China (2.5%) and India (35.7%) and Nepal (27%). Similarly wasting among children is highest in India (21%) followed by Nepal (9.7%) and China (2%). Urban/rural differences and gender differences among children with stunting, wasting, underweight, and overweight and obesity are likely negligible among all three nations.

#### 3.3.3. Prevalence of OWOB Among Childbearing Age Women in China, India, and Nepal

The percentage of women with overweight and obesity is highest in China (34.6%) followed by Nepal (22.2%) and India (20.6%). Urban/rural differences among women with overweight and obesity are likely negligible in all three nations.

## 4. Discussion

Young children and mostly girls are at greater risk of stunting as well as GI parasite infections [4]. A meta-analysis indicated that protozoans like *Cryptosporidium*, *Giardia*, and *Entamoeba* were the leading causes of stunting in children in LMICs [30]. Furthermore, a study among an obese population demonstrated that parasites like adenovirus 36 exert adipogenic action via its effect on adipose tissue in animal models [31]. Interestingly, a recent cross-sectional study in an obese population found no correlation between parasites and nutritional deficiencies or body composition, but did observe a correlation with metabolic syndrome [32]. The various manifestations of malnutrition were historically approached as separate public health concerns. However, the contemporary understanding acknowledges the intrinsic interconnectedness of undernutrition and overnutrition, thereby necessitating the implementation of integrated double-duty actions that simultaneously address multiple dimensions for effective policy solutions. Accompanied with the parasitic infections, the DBM turns into a Triple Burden of Malnutrition, which is the most important contributing factor for the economic burden in LMICs [33].

China has the highest per capita income, which is 10 times that of Nepal, and 4.5 times that of India. Furthermore, China leads in life expectancy (76.5 years), followed by Nepal (70.2 years) and India (68.2 years) (Table 1). The under-5 mortality follows a reverse pattern, with China exhibiting the lowest rate (9.2), followed by Nepal with an over three times higher rate (33.4) and India with an over four times higher rate (38.7) (Table 1). China also maintains the largest proportion of urban population (58%), while the availability of domestically produced food is highest in India (144.4 index) (Table 1). Undernutrition is least prevalent in China (wasting = 2.0%, underweight = 2.5%), followed by Nepal (wasting = 9.7%, underweight = 27.0%) and India (wasting = 21.0%, underweight = 35.7%) (Table 2). Stunting is highly prevalent in India (38.4%) and Nepal (35.8%), with over one-third of children being affected, but less so in China (8.1%). Notably, the rural/urban difference for stunting is highest in China and almost negligible in the other countries. Conversely, OWOB is more common among school children in China (11.5%), compared to India (2.1%) and Nepal (1.2%) (Table 2). In all three countries, the prevalence of OWOB among children (Table 2) is substantially lower than among women (Table 3). Among women, the prevalence of OWOB is highest in China (34.6%), followed by Nepal (22.2%) and India (20.6%) (Table 3).

In general, undernutrition is less common, but OWOB is more common in children in nations with a stronger economic standing (Table 1 and Table 2). This is especially true in China, which has the greatest income level (Table 1) and a significantly higher prevalence of OWOB than underweight, compared to Nepal and India (Table 2). School children and women of childbearing age show significant rural/urban disparities in the incidence of these outcomes (Table 2 and Table 3). Stunting is more prevalent in schoolchildren in rural regions than in urban environments, while no gender differences are observed in the incidence of underweight and OWOB (Table 2). In general, countries with higher per capita incomes have higher rates of OWOB among school children and women of childbearing age than those with lower incomes (Table 1, Table 2 and Table 3). This supports the body of research showing a link between obesity and higher income levels [18,23]. Apart from women of childbearing in China, where the difference is minimal, the prevalence of OWOB is consistently higher in urban than in rural populations (Table 3). This disparity may be due to the greater exposure of urban dwellers to obesogenic factors, such as higher energy density foods and more sedentary lifestyles [17,22]. Remarkably, recent national data in China show that the prevalence is the same for lactating women in rural and urban settings [16].

The effectiveness of the few policies that have been put in place to concentrate on the prevention and control of obesity has not yet been assessed. China has more obesity-related regulations and intervention initiatives than the other two countries combined, yet it also has the worst obesity problem [16,17]. However, over the past 20 years, all three countries have not been able to prevent or address the sharp rise in OWOB. Key factors associated with obesogenic environments are not adequately prevented or controlled. When creating policies and initiatives to combat the obesity epidemic, undernutrition and parasitic infections, and genetic and psychological elements must also be considered for effective implementation and outcome [29]. China and India are now the major economic power of the world and this Triple Burden of Malnutrition (DBM plus parasitic infections) may impose serious health issues consequently accompanied with economic burden to both nations. This directly hinders their economic growth, demanding a substantial reformation of the public health sector.

Many community-level and ground-level measures like awareness, education regarding sanitation and hygiene, regulation of junk and empty calorie foods, the gadgets-to-ground concept among children, a mid-day meal in rural schools for the children, and proper consultations regarding nutrition and infant health with pregnant and lactating women can help to limit undernutrition, overnutrition, and parasitic infections. Although these low-level measures can be effective, national-level measures like government policy reform, strict implementation and regulation, nationwide awareness and education, mass deworming among children, and—if needed—the formation of new laws by the legislative board can be much more effective and long-lasting to combat the Triple Burden of Malnutrition. More research on parasitic infections, overweight and obesity, and underweight and undernutrition could provide a better way of solving this issue in South Asian low- and middle-income countries.

This study has some limitations. First, the topic is narrow, specific, and less researched as limited research articles were obtained during this study. Another major limitation of this review is the language barrier. Many research articles related to DBM and parasitic infections are published in languages other than English which were excluded in our review. This prevented us from accessing a substantial number of research articles and publications related to the DBM and parasitic infections in LMICs. This scoping review aims to identify relevant evidence that meets pre-determined criteria regarding the topic, field, context, concept, and issue under review. Thus this review lacks statistical significance testing.

## 5. Conclusions

The available literature and data showed that the prevalence of DBM has been increasing worldwide with a more rapid increase in South Asia. National economic development is positively associated with an increasing DBM. The DBM along with parasitic infections can lead to a devastating situation called the Triple Burden of Malnutrition in South Asian LMICs. This issue can add an additional economic burden that directly and indirectly hinders the overall economic development due to poor cognitive development in children, and poor work performance and absenteeism in adults. A lack of awareness regarding hygiene and sanitation, and a lack of policies regarding wastage management and dumping are possible causes of high parasitic infections in LMICs. All these factors must be considered when formulating and implementing national policies and programs to address the DBM and parasitic infections simultaneously.

## Figures and Tables

**Table 1:** Economic and demographic characteristics of China, India, and Nepal.

Country	Income Level	GDP per Capita (USD)	Life Expectancy (Year)	Population (Thousand)	Urban Population (%)	Food Production Index	Under-5 Mortality
China	Upper/middle	8827.0	76.5	1,386,395.0	58.0	139.0	9.2
India	Lower/middle	1942.1	68.2	1,339,180.1	33.6	144.4	38.7
Nepal	Low	849.0	70.2	29,305.0	19.3	137.8	33.4

Data source: World Development Indicators and World Bank, 2023. Bank, W. (2023). Ref [34].

**Table 2:** Prevalence (%) of under- and overnutrition among children with their rural/urban and gender (boy/girl) differences.

Country	Population	Stunting (%)	Wasting (%)	Underweight (%)	OWOB (%)
China	Overall	8.1	2.0	2.5	11.5
	Rural/urban	2.7	1.6	1.8	1.0
	Boy/girl	1.2	1.0	1.1	1.3
India	Overall	38.4	21.0	35.7	2.1
	Rural/urban	1.3	1.1	1.3	0.6
	Boy/girl	1.0	1.1	1.0	1.0
Nepal	Overall	35.8	9.7	27.0	1.2
	Rural/urban	1.3	1.1	1.3	0.6
	Boy/girl	1.0	1.0	1.0	1.4

Data source: National Health and Demographics Survey Report: China (Yu et al., 2017 [16]), India (IIPS/India and ICF, 2017 [24]), and Nepal (MOH/Nepal et al., 2017 [28]).

**Table 3:** Prevalence (%) of overweight/obesity among women of childbearing age (15–49 years) and their differences in rural/urban region.

Country	Population	Overweight (%)	Obesity (%)	OWOB (%)
China	Overall	25.4	9.2	34.6
	Rural/urban	1.0	1.0	1.0
India	Overall	15.5	5.1	20.6
	Rural/urban	0.5	0.3	0.5
Nepal	Overall	17.1	5.1	22.2
	Rural/urban	0.7	0.3	0.6

Data source: National Health and Demographics Survey Report: China (Yu et al., 2017 [16]), India (IIPS/India and ICF, 2017 [24]) and Nepal (MOH/Nepal et al., 2017 [28]).

## Data Availability

The original contributions presented in this study are included in the article/Supplementary Material. Further inquiries can be directed to the corresponding author.

## Supplementary Materials

The following supporting information can be downloaded at: https://www.mdpi.com/article/10.3390/nu17213494/s1, Table S1: PRISMA 2020 Checklist (Page et al., 2021) [10].
